# The application of drug-coated balloon to *in situ* short and long lesions of large coronary artery vessels

**DOI:** 10.3389/fcvm.2026.1871063

**Published:** 2026-08-20

**Authors:** Xigui Yan, Mingwei Wang

**Affiliations:** Department of Cardiology, Affiliated Hospital of Hangzhou Normal University, Hangzhou, China

**Keywords:** coronary artery disease, drug-coated balloon, *in situ* long lesions of large coronary artery vessels, *in situ* short lesions of large coronary artery vessels, percutaneous coronary intervention

## Abstract

**Objective:**

To observe the efficacy of drug-coated balloon (DCB) in the treatment of *in situ* short (reference diameter ≥2.8 mm) and long lesions (reference diameter ≥2.8 mm, length ≥20 mm) of large coronary artery vessels.

**Methods:**

A total of 126 patients who were diagnosed and treated in the Department of Cardiology, Affiliated Hospital of Hangzhou Normal University from April 2021 to December 2024 were selected, and all of them were treated with DCB for *in situ* primary coronary artery lesions. Computer-based quantitative coronary angiography analysis software was used to analyze the coronary angiography images of included patients obtained at three different time points; pre-interventional procedure, immediate post-interventional procedure, and follow-up. The selected patients were divided into two groups according to the length of target vessel lesions, namely the lesion length <20 mm group (*n* = 68) and the lesion length ≥20 mm group (*n* = 58). The inter-group comparison was performed to evaluate the efficacy of DCB in long *in situ* coronary artery lesions.

**Results:**

The average length of the target vessel lesion in the included cases was 16.64 ± 5.65 mm, and the reference diameter of the target vessel lesion was 3.19 ± 0.35 mm. During follow-up of 14.80 ± 8.55 months, none of the patients suffered acute myocardial infarction or severe arrhythmia, cardiogenic shock, and stroke. None of the cases showed revascularization of the target vessel at follow-up, and the late lumen loss during follow-up was −0.03 ± 0.23 mm. There was no significant difference in late lumen loss between the two groups of patients with different lesion lengths [−0.03 ± 0.21 mm vs. −0.02 ± 0.25 mm, *P* = 0.803].

**Conclusions:**

DCB is safe and effective in treating *in situ* short and long lesions of large coronary artery vessels, and it can reduce the late lumen loss in coronary target lesions.

## 1 Introduction

With the increase in the incidence rate of coronary heart disease and the development of percutaneous coronary intervention (PCI), the number of PCI interventional procedures in China has showed an average annual consistent growth of 15%–20% in the past 10 years (1). At present, drug-eluting stent (DES) implantation is still the most common method of PCI (2). The anti-proliferative drugs carried by DES can inhibit proliferation of the smooth muscle and endothelial cells. After DES implantation, polymers are required to allow binding of the anti-proliferative drugs to the surface of the stent, and the anti-proliferative drug should be slowly released at a certain speed. However, the polymer coating and metal residues of stents can induce inflammatory reactions, which in turn promote the development of neoatherosclerosis (3). Drug-coated balloons (DCBs) also carry anti-proliferative drugs. When a DCB is inflated, the anti-proliferative drugs are released locally at a single timepoint and can be absorbed quickly to achieve a long-term inhibitory effect. Compared with DES, which is associated with reduction of polymeric coatings and metal materials, DCB helps to reduce intimal inflammation, inhibit intimal hyperplasia, and reduce the risk of thrombosis (4). Therefore, DCB has a therapeutic effect on in-stent restenosis (ISR), primary coronary artery disease, and bifurcation disease. In addition, since the antiplatelet duration after DCB treatment is short (dual antiplatelet therapy for 1–3 months), it is also applicable to patients with renal insufficiency, advanced age, and high-risk bleeding tendency (5).

In the clinic, DCB has also been gradually applied to treat *de novo* lesions of coronary arteries. However, due to the short follow-up time and limited reports of relevant studies, its efficacy is debatable (6). There is a lack of relevant research on DCB for the treatment of *de novo* long lesions of large coronary arteries. Hence, we conducted a retrospective study in patients with *de novo* coronary artery lesions at the Affiliated Hospital of Hangzhou Normal University. The aim of this study is to explore the efficacy of DCB in the treatment of *de novo* coronary artery lesions, including diffuse long coronary artery lesions, so as to provide more data for the clinical application of DCB.

## 2 Material and methods

### 2.1 General information

From April 2021 to December 2024, 126 patients diagnosed and treated in the Department of Cardiology of the Affiliated Hospital of Hangzhou Normal University were selected.

Inclusion criteria were as follows: ① *de novo* lesions of large coronary artery vessels treated with DCB, and completion of coronary angiography (CAG) reexamination; ② in accordance with the recommendations of the international DCB consensus group and the Chinese expert consensus on the clinical application of DCB (7), large vessel disease (LVD) of the coronary artery defined as the reference diameter of the diseased vessel ≥2.8 mm.

Exclusion criteria were as follows: ① Patients treated with DCB due to ISR ② Patients with salvage stent implantation during DCB. All patients with coronary heart disease signed the informed consent form before the interventional procedures.

### 2.2 Methods

Baseline data of patients, including age, sex, body mass index (BMI), systolic blood pressure (SBP), diastolic blood pressure (DBP), heart rate (HR), HbA1c, blood lipid, creatinine (CRE), uric acid (UA), left ventricular ejection fraction (LVEF), family history of coronary heart disease, previous smoking history, hypertension, diabetes, PCI history, type of coronary heart disease, baseline characteristics during DCB, and postoperative antithrombotic strategy, were collected.

#### 2.2.1 DCB interventional treatment process

The interventional procedures recommended in the “Drug-Coated Balloons for Coronary Artery Disease: Third Report of the International DCB Consensus Group”, which was based on the International DCB Application Consensus, were performed. All patients received standard dual antiplatelet therapy. Aspirin 100 mg once daily plus clopidogrel 300 mg once daily, followed by 75 mg once daily, or ticagrelor loading dose of 180 mg, followed by 90 mg twice daily. Since satisfaction with lesion pretreatment is directly related to the immediate success rate of DCB application and the long-term treatment effect, we used a pre-dilated balloon to gradually expand the lesion from small to large. Then, special balloons, such as non-compliant balloon, double guidewire balloon, scoring balloon, and cutting balloon, were used for lesion pretreatment. Subsequently, the effect was evaluated after intravenous administration of an appropriate amount of nitroglycerin. The following conditions were fulfilled after lesion pretreatment: residual stenosis <30%, TIMI blood flow grade 3, and non-flow limiting dissection. If the above requirements were met, a DCB with a balloon to vessel diameter ratio of 1:1 ratio was selected, and the length was 2–3 mm longer than both ends of the treated lesion to prevent lumen loss. The DCB was expanded at a pressure of 7–14 atm for 30–60 s. The success criteria after DCB treatment were the same as those for the aforementioned pretreatment.

#### 2.2.2 Follow-up and index observation

CAG images of all selected patients were collected at the time of pre-interventional procedure and immediate post-interventional procedure, and follow-up. Then QCA analysis software was used to measure the lesion data: length (mm), reference vessel diameter (mm), minimal luminal diameter (MLD), diameter stenosis (DS%), area stenosis (AS%), immediate gain (acute gain) = immediate postoperative MLD—preoperative MLD, late lumen loss (LLL) = immediate postoperative MLD—postoperative follow-up MLD, and net gain = postoperative follow-up MLD—preoperative MLD. The QCA analysis was conducted independently by two experienced imaging physicians who were blinded to the patients' clinical data and procedural outcomes. The interventional operators did not participate in the angiographic measurements. In cases of inter-observer discrepancy, a consensus was reached through discussion or by consulting a third senior investigator. Each lesion was measured three times, and the average values were used for final analysis. After measuring the relevant data at three different time points, a pre-and-post comparison was performed to evaluate the efficacy of DCB. According to the length of target vessel lesions, the selected patients were divided into two groups, namely the lesion length <20 mm group and the lesion length ≥20 mm group, and data in the two groups were compared to evaluate the efficacy of DCB in the treatment of long *in situ* coronary artery lesions. Endpoints included target lesion revascularization (TLR) and major adverse cardiovascular events (MACE). TLR was defined as follows: follow-up CAG re-examination indicating that the target lesion DS% was ≥50% with myocardial ischemia-related symptoms or no ischemia symptoms but the DS% was ≥70%, and the target lesion needed retreatment. MACE was defined as follows: acute myocardial infarction, severe arrhythmia, and cardiac death.

Quantitative flow ratio (QFR) is a non-invasive, angiography-derived computational technique that evaluates the functional significance of coronary stenosis without requiring pressure wires or hyperemic agents. In this study, QFR analysis was performed using the AngioPlus system (Pulse Medical Imaging Technology, Shanghai, China). A single, high-quality angiographic frame at end-diastole was selected for vessel contour extraction. Normal proximal and distal segments were designated as reference points, and the corresponding pressure drops were computed utilizing validated algorithms. The QFR measurement was conducted by a certified technician who was blinded to the patients' clinical and procedural outcomes. All computed QFR data were subsequently verified by a second independent, certified technician.

### 2.3 Statistical analysis

SPSS 11.5 software was used for statistical analysis. measurement data are shown as ±*s,* and count data are shown as the number of cases and rates. Reference blood vessel diameter, minimum lumen diameter, degree of DS%, degree of AS%, and other indicators were compared among pre-interventional procedure, immediate post interventional procedure, and during follow-up. LSD method was used for pairwise comparison. Group comparison of lesion length was performed by multivariate analysis of variance (Hotelling's Trace), and *p* < 0.05 was considered statistically significant.

## 3 Results

### 3.1 Baseline data of the patients

A total of 126 patients were enrolled in this study. The patient age ranged from 38 years to 87 years (average age, 63.25 ± 10.58 years), with 97 males (77.0%) and 29 females (23.0%). The patient's medical history, type of coronary heart disease, blood pressure, heart rate, BMI, LVEF, glycosylated hemoglobin, blood lipids, renal function, and other related indicators are presented in Table 1.

**Table 1:** Baseline data of the patients.

Project	Result
Age (years)	63.25 ± 10.58
Gender (male/female, number)	97/29
Systolic blood pressure (mmHg)	126.10 ± 13.27
Diastolic blood pressure (mmHg)	71.06 ± 9.29
Rate (times/min)	66.94 ± 7.76
BMI (kg/m^2^)	24.29 ± 2.97
Glycosylated hemoglobin (%)	6.40 ± 1.11
Triglycerides (mmol/L)	1.45 ± 0.74
Total cholesterol (mmol/L)	3.59 ± 0.91
High density lipoprotein (mmol/L)	1.06 ± 0.26
Low density lipoprotein (mmol/L)	2.04 ± 0.67
Creatinine (µmol/L)	83.67 ± 44.04
Uric acid (µmol/L)	372.98 ± 90.78
LVEF	0.59 ± 0.06
History of PCI [number (%)]	50 (39.68)
Hypertension [number (%)]	91 (72.22)
Cigarettes [number (%)]	62 (49.21)
Quit smoking [number (%)]	19 (30.65)
Diabetes [number (%)]	38 (30.16)
Types of CAD	
Unstable angina [number (%)]	62 (49.21)
Stable angina[number (%)]	37 (29.37)
NSTEMI^a^ [number (%)]	18 (14.29)
STEMI^b^[number (%)]	9 (7.14)

a NSTEMI is non-ST-segment elevation myocardial infarction.

b STEMI is ST-segment elevation myocardial infarction.

### 3.2 Baseline characteristics of patients who received a DCB

All 126 lesions in the enrolled patients were treated with DCB alone without remedial stent placement. The DCB diameter was 3.0∼3.5 mm. 136 predilatation balloons were used in 126 lesions. With respect to the selection of predilatation balloon, a non-compliant balloon was used in 13.24% (18/136) of cases, double guidewire balloon was used in 29.41% (40/136) of cases, scoring balloon was used in 27.21% (37/136) of cases, and cutting balloon was used in 30.15% (41/136) of cases. The incidence rate of coronary artery dissection following DCB application was 7.94% (10/126), comprising 4 type A, 3 type B, and 3 type C dissections. The management of the 3 type C dissections was guided by physiological assessment. These target lesions were located in the left circumflex artery (LCX, *n* = 2) and the left anterior descending artery (LAD, *n* = 1). Post-procedural quantitative flow ratio (QFR) was calculated to assess the functional severity of the dissections, yielding values of 1.00, 0.96, and 0.89, respectively. Given that the distal flow remained physiologically unrestricted, a conservative strategy was adopted without salvage stent implantation. At the angiographic follow-up, all 3 cases demonstrated morphological vessel healing with no target lesion revascularization (TLR) or residual flow-limiting stenosis (Table 2).

**Table 2:** Baseline characteristics.

Project	Result
Lesion sites treated with DCB [number (%)]	
Left anterior descending branch	56 (44.44)
Left circumflex artery	28 (22.22)
Right coronary artery	32 (25.40)
Bifurcation lesions of left main	10 (7.94)
Type of pre-expanded balloon [number (%)]	
non-compliant balloon	18 (13.24)
Double guidewire balloon	40 (29.41)
Neuron specific enolase	37 (27.21)
Cutting balloon	41 (30.15)
Diameter of DCB (mm)	3.09 ± 0.27
Length of DCB (mm)	21.71 ± 5.56
Expansion time of DCB (s)	58.10 ± 8.07
Expansion pressure of DCB (atm)	10.11 ± 2.17
Dissection post-procedure [number (%)]	10 (7.94)
A type	4
B type	3
C type	3

### 3.3 Baseline characteristics after grouping patients according to the lesion length

The baseline data of the lesion length <20 mm group (*n* = 68) and the lesion length ≥20 mm group (*n* = 58) were compared. As shown in Table 3, there was no significant difference in the baseline data between the two groups (*P* > 0.05).

**Table 3:** The baseline data of two groups ( (lesion length <20 mm or ≥20 mm).

Project	lesion length <20 mm (*n* = 68)	lesion length ≥20 mm (*n* = 58)	t value	*P*	95% confidence interval
Age (years)	64.65 ± 9.96	61.62 ± 11.13	1.611	0.110	(−6.746, 0.693)
Systolic blood pressure (mmHg)	126.51 ± 13.99	125.60 ± 12.48	0.383	0.702	(−3.800, 5.622)
Diastolic blood pressure (mmHg)	69.96 ± 9.35	72.36 ± 9.12	−1.456	0.148	(−5.677, 0.865)
Rate (beats/min)	66.75 ± 7.91	67.16 ± 7.64	0.291	0.772	(−3.161, 2.351)
BMI (kg/m^2^)	24.38 ± 3.07	24.19 ± 2.88	0.353	0.725	(−0.867, 1.244)
Glycated hemoglobin (%)	6.42 ± 1.08	6.38 ± 1.15	0.229	0.819	(−0.349, 0.440)
Triglycerides (mmol/L)	1.44 ± 0.60	1.46 ± 0.89	0.164	0.870	(−0.285, 0.242)
Total cholesterol (mmol/L)	3.65 ± 0.93	3.52 ± 0.88	0.798	0.427	(−0.192, 0.451)
High density lipoprotein (mmol/L)	1.09 ± 0.27	1.03 ± 0.25	1.089	0.278	(−0.042, 0.143)
Low density lipoprotein (mmol/L)	2.08 ± 0.68	1.99 ± 0.67	0.765	0.446	(−0.147, 0.331)
Creatinine (*μ*mol/L)	87.44 ± 56.10	79.25 ± 22.71	1.040	0.300	(7.388, 23.759)
Uric acid (μmol/L)	364.53 ± 96.21	382.88 ± 83.72	1.132	0.260	(−50.430, 13.730)
LVEF	0.59 ± 0.06	0.59 ± 0.06	0.220	0.826	(−0.198, 0.025)
Perioperative drug use [%(*n*/N)]					
DAPT	100 (68/68)	100 (58/58)		1.000	
Statins	100 (68/68)	100 (58/58)		1.000	

### 3.4 Clinical follow-up results

The follow-up period was 14.80 ± 8.55 months. During the follow-up period after DCB treatment, acute myocardial infarction, severe arrhythmia, cardiac death, or stroke did not occur. There were no cases of revascularization of target vessels in all patients at follow-up.

Comparison of target vascular indexes at the time of pre-interventional procedure, immediate post-interventional procedures, and during follow-up:

1. There was no significant difference in the reference vessel diameter (mm) at the time of pre-interventional procedure, immediate post- interventional procedure, and during follow-up (*F* = 0.358, *P* = 0.700).
2. There was a significant difference in MLD (*F* = 1,007.161, *P* < 0.001). Among them, the MLD at the time of immediate post-interventional procedure was much higher than that at the time of pre-interventional procedure, and the difference was statistically significant (MD = −1.667, *P* < 0.001); and the MLD at follow-up was much higher than that at the time of pre-interventional procedure, and the difference was statistically significant (MD = −1.693, *P* < 0.001). There was no significant difference in the MLD between immediate post- interventional procedure and at follow-up (MD = −0.026, *P* = 0.550).
3. The degree of DS% was significantly different (*F* = 1,256.709, *P* < 0.001). Among them, the immediate postoperative index was significantly lower than that at the time of pre-interventional procedure (MD = 51.548, *P* < 0.001), and the follow-up index was significantly lower than that at the time of pre-interventional procedure (MD = 52.238, *P* < 0.001). There was no significant difference between the immediate postoperative period and follow-up (MD = 0.690, *P* = 0.564).
4. There was a significant difference in the degree of AS% (*F* = 1,029.593, *P* < 0.001). The degree of AS% at the time of immediate post-interventional procedure was much smaller than that at the time of pre-interventional procedure, and the difference was statistically significant (MD = 53.810, *P* < 0.001). The degree of AS% at the time of follow-up was much less than that at the time of pre-interventional procedure, and the difference was statistically significant (MD = 54.921, *P* < 0.001). There was no significant difference between the immediate postoperative period and follow-up (MD = 1.111, *P* = 0.422) (Table 4).

**Table 4:** Results of QCA.

Index	$\bar{x}\pm s$	95% confidence interval
Reference vessel diameter (mm)		
Pre	3.19 ± 0.35	(3.128, 3.250)
Immediate post-procedure	3.22 ± 0.34	(3.155, 3.276)
Follow-up	3.23 ± 0.36	(3.162, 3.288)
*F* value	0.358	
*P* value	0.700	
MLD (mm)		
Pre	0.85 ± 0.37^b^	(0.784, 0.916)
Immediate post-procedure	2.52 ± 0.33^a^	(2.459, 2.576)
Follow-up	2.54 ± 0.32^a^	(2.487, 2.600)
*F* value	1,007.161	
*P* value	<0.001	
Degree of diameter stenosis (%)		
Pre	73.10 ± 11.61^b^	(71.048, 75.143)
Immediate post-procedure	21.55 ±± 8.26^a^	(20.092, 20.004)
Follow-up	20.86 ± 8.19^a^	(19.414, 22.300)
*F* value	1,256.709	
*P* value	<0.001	
Degree of area stenosis (%)		
Pre	91.59 ± 5.19^b^	(90.678, 92.497)
Immediate post-procedure	37.78 ± 13.03^a^	(35.480, 40.076)
Follow-up	36.67 ± 12.86^a^	(34.400, 38.934)
*F* value	1,029.593	
*P* value	<0.001	

a compared with pre-procedure: *P*  < 0.05.

b compared with post-procedure: *P* < 0.05.

**Figure 1:**
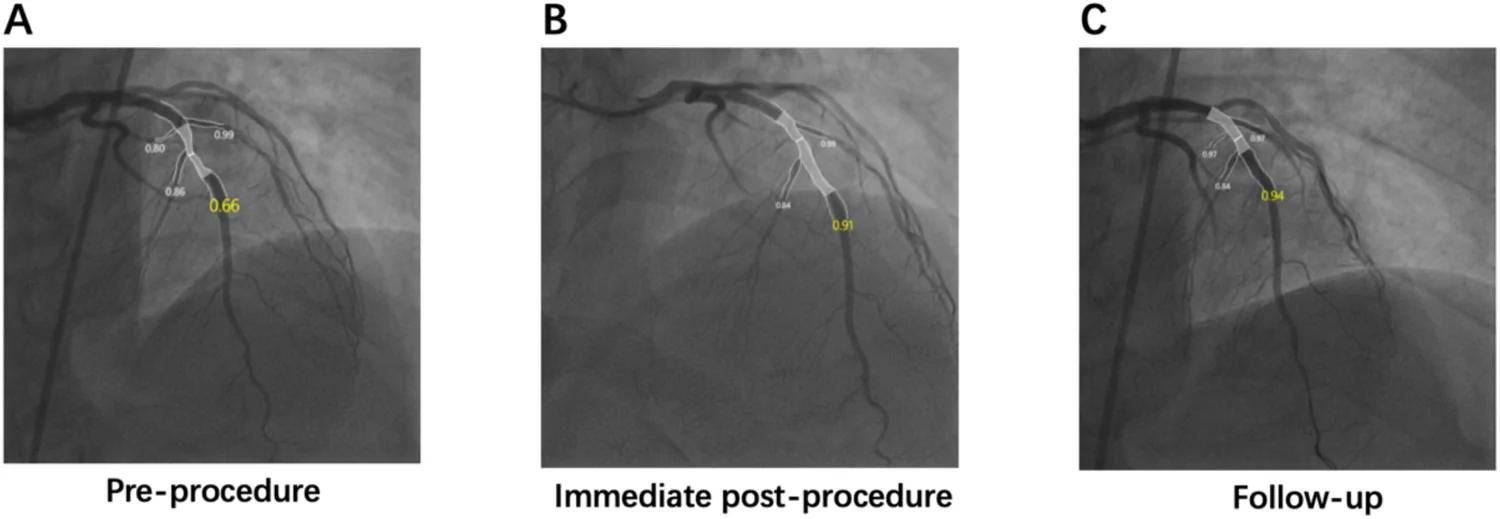
Representative angiographic and quantitative flow ratio (QFR) outcomes of a *de novo* coronary lesion treated with a drug-coated balloon (DCB). **(A)** Pre-procedure QFR analysis reveals a significant stenosis in the LAD with a baseline QFR of 0.66. **(B)** Immediate post-procedure QFR analysis demonstrates luminal improvement following DCB optimization, with the QFR increasing to 0.91, indicating unrestricted distal physiological flow. **(C)** Follow-up QFR analysis demonstrates sustained vessel patency and further late lumen enlargement, with a QFR of 0.94, in the absence of target lesion revascularization or residual flow-limiting stenosis.

**Figure 2:**
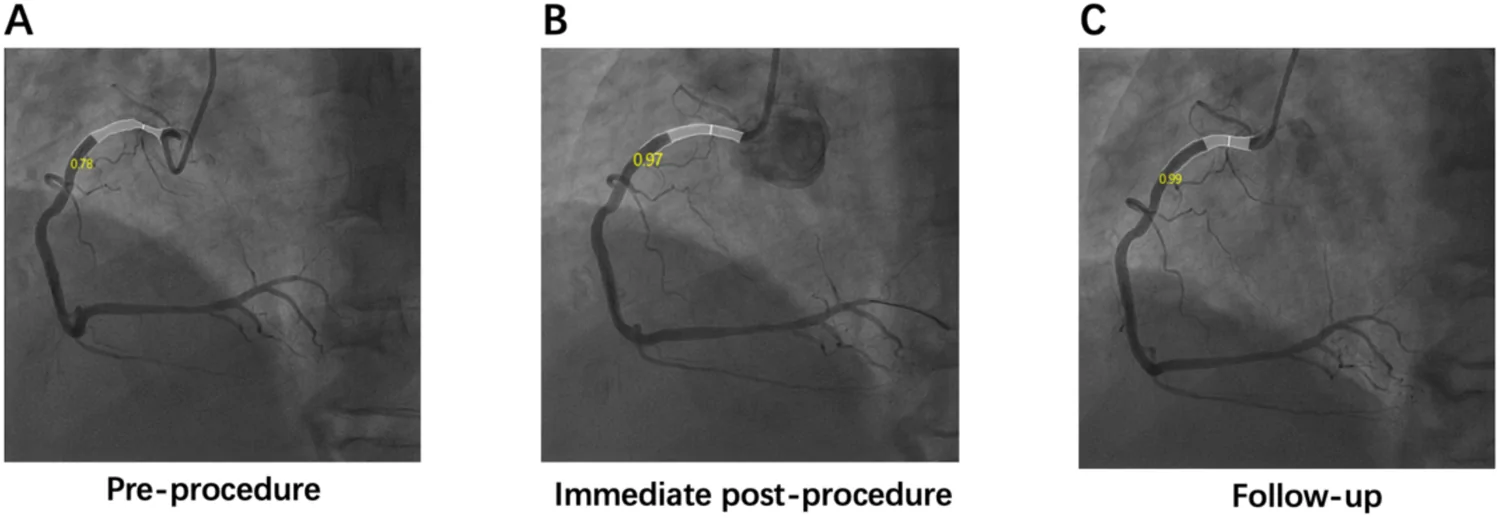
Serial angiographic and QFR outcomes of a diffuse long lesion in the RCA treated with DCB, demonstrating late lumen enlargement. **(A)** Pre-procedure QFR analysis shows a severe stenosis with a baseline QFR of 0.78. **(B)** Immediate post-procedure QFR increases to 0.97 following DCB treatment. **(C)** Follow-up QFR analysis demonstrates sustained vessel patency and further physiological improvement with a QFR of 0.99, indicative of positive late lumen remodeling.

**Figure 3:**
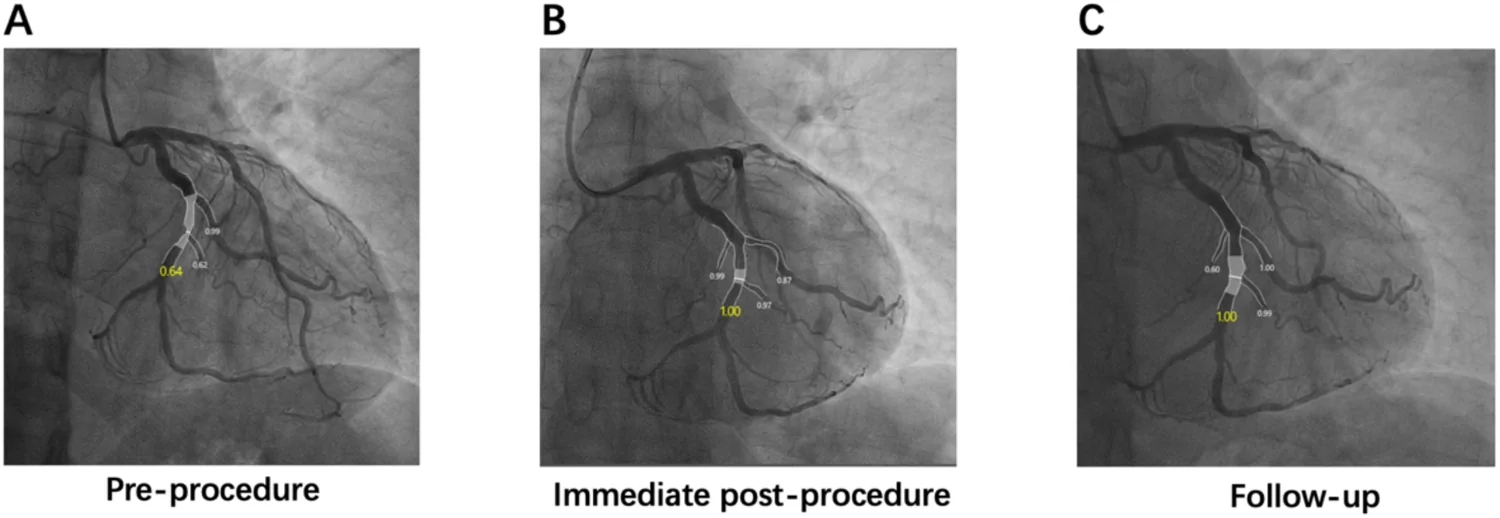
Serial angiographic and QFR outcomes of a LCX lesion complicated by dissection, managed conservatively. **(A)** Pre-procedure QFR analysis reveals a severe stenosis with a baseline QFR of 0.64. **(B)** Immediate post-procedure QFR analysis shows a visible dissection after DCB application; however, the corresponding QFR is 1.00, indicating unrestricted distal flow, which supported the deferral of salvage stenting. **(C)** Follow-up QFR analysis demonstrates complete morphological healing of the dissection and further lumen enlargement, maintaining a QFR of 1.00.

Representative cases demonstrating the serial angiographic and physiological (QFR) outcomes of *de novo* coronary lesions treated with DCB are illustrated in Figures 1–3. Figure 1 illustrates a standard treatment course in the left anterior descending artery (LAD). Figure 2 demonstrates the treatment of a diffuse long lesion in the right coronary artery (RCA) exhibiting late lumen enlargement during follow-up. Figure 3 highlights a case in the left circumflex artery (LCX) complicated by a post-procedural dissection. Conservative management was adopted based on an unrestricted post-procedural QFR of 1.00, which subsequently demonstrated complete morphological healing and further lumen enlargement at angiographic follow-up.

#### 3.4.1 Group comparison of lesion length

The comparison of indicators between the two groups is shown in Table 5. The reference vessel diameter, immediate postoperative diameter stenosis, immediate postoperative area stenosis, follow-up diameter stenosis, and follow-up area stenosis were higher in the lesion length ≥20 mm group than in the lesion length <20 mm group; and the differences were statistically significant (*p* < 0.05). There was no significant difference in the other indexes between the two groups (all *p* > 0.05).

**Table 5:** Comparison of target vessel-related indexes in patients with different lesion length groups ($\bar{x}\pm s$).

Project	lesion length < 20 mm	lesion length ≥ 20 mm	*F*	*P*
Postoperative reference vessel diameter (mm)	3.11 ± 0.30	3.28 ± 0.38	7.363	0.008
Postoperative MLA (mm)	0.89 ± 0.31	0.80 ± 0.43	1.841	0.177
Postoperative diameter stenosis (%)	71.51 ± 9.41	74.95 ± 13.61	2.776	0.098
Postoperative area stenosis (%)	91.13 ± 4.66	92.12 ± 5.68	1.150	0.286
Post reference vessel diameter (mm)	3.15 ± 0.31	3.30 ± 0.36	5.909	0.016
Immediate postoperative MLA (mm)	2.53 ± 0.32	2.50 ± 0.35	0.327	0.568
Immediate postoperative diameter stenosis (%)	19.44 ± 7.96	24.02 ± 7.98	10.328	0.002
Immediate postoperative area stenosis (%)	34.53 ± 12.63	41.59 ± 12.56	9.823	0.002
Immediate inner diameter obtained (mm)	1.64 ± 0.36	1.70 ± 0.57	0.452	0.503
Follow-up reference vessel diameter (mm)	3.16 ± 0.31	3.30 ± 0.40	4.474	0.036
Follow-up MLA (mm)	2.56 ± 0.30	2.52 ± 0.34	0.602	0.439
Follow-up diameter stenosis (%)	18.88 ± 7.61	23.17 ± 8.30	9.159	0.003
Follow-up area stenosis (%)	33.63 ± 11.97	40.22 ± 13.04	8.738	0.004
Late luminal loss (mm)	−0.03 ± 0.21	−0.02 ± 0.25	0.063	0.803
Net inner diameter obtained (mm)	1.67 ± 0.37	1.72 ± 0.58	0.291	0.590

## 4 Discussion

In the past few decades, coronary intervention has experienced the era of “from nothing to something”. DES implantation is still regarded as the standard treatment of coronary intervention. However, the presence of permanent residues in the coronary artery leads to a high risk of ISR. On the premise of ensuring the safety of patients and effectiveness of treatment, interventionists and patients with coronary heart disease should urgently realize the “from presence to absence” of coronary intervention therapy; i.e., to reduce or even eliminate the legacy of instruments. DCB treatment is considered to benefit patients with ISR (Evidence Grade IA) (8), In 2020, the international consensus on the use of DCB for the treatment of coronary artery disease pointed out that DCB may be suitable for the treatment of *in situ* coronary artery disease in addition to ISR (6). It is worth noting that for *de novo* small vessel lesions, two previous large-scale trials showed that the efficacy of DCB was not inferior to that of second-generation DES (9, 10). With the widespread development of DCB, clinicians are more flexible in the choice of indications. DCB may be more advantageous than DES in some special patients, such as patients with coronary heart disease and diabetes. The incidence of complications after coronary intervention in these patients is high. DCB may be superior to DES, but further studies are needed to confirm this finding. For patients with high bleeding risk, dual antiplatelet therapy is recommended for 4 weeks after DCB alone treatment of *de novo* lesions. In this manner, the incidence of bleeding events is relatively low, which makes DCB treatment more advantageous. For coronary bifurcation lesions, ESC guidelines recommend that DES implantation in the main branch and DCB placement in the contralateral branch may be superior to angioplasty. For patients with acute coronary syndrome, the PEPCAD NSTEMI and REVELATION studies showed that DCB was not inferior to stent implantation (11, 12). Some young patients (<45 years old) with coronary heart disease are more inclined to receive DCB treatment in the absence of clear contraindications. Indeed, PCI in small vessels only accounts for approximately 35% of all coronary interventions (13). In addition, guidelines indicate that DCB treatment appears to be safe and effective for *de novo* lesions of large coronary vessels (≥2.8 mm), but there is still a lack of adequate evidence.

In this study, all the included coronary artery lesions fulfilled the relevant criteria for *de novo* large vessel lesions. The length of target vessel lesions was 16.64 ± 5.65 mm, and the reference diameter was 3.19 ± 0.35 mm. The follow-up time was 14.80 ± 8.55 months. The results showed that the MLD in the patients immediately after surgery and at follow-up was significantly higher than that before surgery, and there was no significant difference between the MLD at follow-up and immediately after surgery. Even in some patients, the MLD at follow-up was larger than that immediately after surgery. The degree of luminal stenosis in patients during the immediate post- interventional procedure and at follow-up was much less than that during the pre-interventional procedure, and there was no statistical difference in luminal stenosis at follow-up compared with the immediate post-interventional procedure. In addition, during the follow-up, acute myocardial infarction, severe arrhythmia, cardiac death, stroke, severe bleeding, and other events did not occur. None of the cases showed TLR, and the LLL was −0.03 ± 0.23 mm. All these results indicate that DCB is safe and effective in the treatment of *de novo* lesions of coronary large vessels with low lumen loss and restenosis rate.

The conclusions of this study are consistent with previous studies on DCB treatment of coronary large vessel lesions *in situ* (11, 14, 15), and this study offers the following advantages: First, compared with other studies, this study used more special types of balloons for the pretreatment of lesions. For lesions with heavy plaque load and severe calcification and fibrosis, we often choose a small- diameter balloon to expand step by step in order to reduce the elastic recoil of large vessels and the occurrence of severe dissection. The special balloons used in the enrolled patients included non-compliant balloons (18/136), double guidewire balloons (40/136), scoring balloons (37/136), and cutting balloons (41/136). Such balloons can increase the immediate luminal acquisition of target lesions, reduce the incidence of severe dissection and hematoma, and improve the uptake of antiproliferative drugs in target lesions, thus improving the efficacy of DCB. In a study of DCB for *de novo* lesions of large coronary vessels, 222 patients were enrolled, of whom 45 underwent follow-up CAG. A cutting balloon or scoring balloon was used for pretreatment of 40% of target lesions during interventional procedures, and TLR or MACE was not found during an average follow-up of 10 months (16). Second, for the treatment of target lesions, we have strictly followed the relevant interventional procedures. After pretreatment of target lesions, angiographic results should fulfill the following conditions before DCB treatment, including residual stenosis <30%, TIMI flow grade 3, and non-flow limiting dissection. In this study, it was considered that among the above conditions, it was particularly important whether the distal blood flow of the target diseased vessel reached TIMI flow grade 3. The relevant expert consensus indicates that it may be useful to measure FFR for target lesions to be treated with DCB if dissection or limited lumen expansion occurs after predilatation. An FFR value ≥0.80 may be appropriate. Another animal experiment showed that the dissection with unrestricted blood flow may better promote the absorption of anti-proliferative drugs in a DCB and the expansion of lumen in the late stage. In this study, 3 patients developed type C dissection after pretreatment. However, the distal blood flow was still TIMI grade 3 and the intraoperative QFR value was ≥0.80; thus, salvage stent implantation was not performed. Finally, the different types and processes of drug balloon selection in different cardiovascular treatment centers, the different follow-up time periods, and the difference in the control of coronary heart disease risk factors in patients are highlights of this study.

In this study, the MLD was 2.52 ± 0.34 mm during the immediate post-interventional procedure, the MLD was 2.54 ± 0.32 mm at follow-up CAG, and the LLL was −0.03 ± 0.23 mm. In 59 of the 126 included lesions (52%), MLD was greater during angiographic follow-up than immediately after DCB (2.59 ± 0.27 mm vs. 2.41 ± 0.29 mm). This indicates that there is not only loss of late lumen diameter in these patients, but it is also expanded (positive remodeling), which is consistent with the current mainstream research conclusion. KLEBER et al. (17) found that some patients (69%) showed positive lumen remodeling during drug balloon treatment of *de novo* coronary small vessel lesions (1.75 ± 0.55 mm *vs.*1.91 ± 0.55 mm, *P* < 0.001); and 33% of them had lumen diameter larger than 0.2 mm in the late stage of lesions. Some scholars also reached the same conclusion in the observation of the clinical effect of DCB in the treatment of *de novo* lesions of large coronary artery vessels (18). The mechanism underlying this phenomenon is still unclear. It is mainly believed that the antiproliferative drugs carried by DCB act on vascular smooth muscle cells and endothelial cells, inducing local partial apoptosis and inhibiting their proliferation. It may also be associated with vascular healing and plaque retraction.

In patients with diabetes mellitus or chronic kidney disease, and in the elderly and women, the characteristics of the lesions often include calcification, distortion, angulation, and multi-vessel disease. Therefore, complications, such as dissection, poor stent attachment, slow flow and no- reflow, are prone to occur during PCI for such lesions. This is also an important reason for the occurrence of ISR, TLR, subacute stent thrombosis, and other adverse events after PCI. The prospective PEPCAD I study is the first clinical study of DCB in patients with high-risk small vessel disease. The results showed that DCB alone treatment for patients with long lesions of small coronary vessels can reduce the intraoperative and postoperative complications and LLL, and reduce the incidence of MACE (19). However, there are few studies about the use of DCB in the treatment of *de novo* long coronary artery lesions. In this study, the comparison of the groups with long and short lesions showed that target lesions with lesion length ≥20 mm had larger reference vessel diameter, immediate postoperative diameter, diameter at follow-up, and AS% than lesions with lesion length <20 mm. The difference was statistically significant (*p* < 0.05). However, there was no significant difference in LLL between the two groups (−0.03 ± 0.21 mm *vs.*−0.02 ± 0.25 mm, *P* = 0.803). This indicates that, although limited by the characteristics of diffuse long coronary artery lesions, pretreatment of target vessels in the process of DCB treatment often cannot achieve the dilatation effect as in short lesions. However, during the long-term follow-up, lumen loss in the late stage of long lesions was comparable to that in short lesions, and TLR and MACE were not found. The interventional treatment of diffuse long coronary lesions with DES often necessitates extensive metal coverage. Our current data demonstrate acceptable late lumen loss and procedural safety when utilizing DCB for these extended lesions. While the single-arm design precludes efficacy comparisons with DES, the angiographic outcomes observed in this cohort, combined with the fundamental advantage of avoiding permanent implants across long vascular segments, indicate that DCB represents a viable therapeutic option.

This study has some limitations. As a single-center retrospective study, the number of cases was relatively small. In addition, only data on DCB treatment for coronary macroangiopaplasia were included in this study, without comparison with data related to DES implantation. Therefore, multicenter randomized clinical trials are needed in the future to further evaluate the efficacy of DCB and second-generation DES in the treatment of *de novo* large vessel lesions and long coronary artery lesions.

## Data Availability

The raw data supporting the conclusions of this article will be made available by the authors, without undue reservation.
